# Source rock evaluation of Afowo clay type from the Eastern Dahomey Basin, Nigeria: insights from different measurements

**DOI:** 10.1038/s41598-020-68918-y

**Published:** 2020-07-21

**Authors:** Saeed Mohammed, Mimonitu Opuwari, Salam Titinchi

**Affiliations:** 1 Department of Earth Sciences, Faculty of Natural Sciences, University of the Western Cape, Western Cape, Bellville, South Africa; 2 Department of Chemistry, Faculty of Natural Sciences, University of the Western Cape, Western Cape, Bellville, South Africa

**Keywords:** Ocean sciences, Solid Earth sciences, Energy science and technology

## Abstract

The Cretaceous Afowo Formation in the Eastern Dohamey Basin is characterized by an admixture of lithofacies ranging from sandstones, claystones, shales, clays, sand/shale, and sand/clay intercalations. The sandy facies, a mix of sandstone, clay, shale, and intercalations, contain biodegraded hydrocarbons while the shales and claystones that underlie it are rich in organic matter. The hydrocarbon-bearing interval is commonly referred to as the oil sand or tar sand. In this study, Afowo clay type underlying an outcrop of the oil sand was appraised for its hydrocarbon potential with loss on ignition, thermogravimetry, and rock evaluation pyrolysis. Results obtained from loss on ignition showed that total organic matter content, a proxy to total organic carbon, for the Afowo clay type ranged from 9.410 to 38.750 wt%. The organic maturation temperature (Tmax) was determined using both thermogravimetry and rock evaluation pyrolysis (Rock–Eval). Thermogravimetric analysis produced reliable Tmax within the range of 417–424 °C for all the samples. The results from rock evaluation pyrolysis on the same samples showed that total organic carbon ranged from 0.81 to 18.46 wt% with Tmax ranging from 417 to 424 °C. It was not possible to determine Tmax for one of the samples with Rock–Eval due to a small S2 value (0.22 mg Hc/g). The variations in organic matter contents from loss on ignition agree with total organic carbon computed from rock evaluation pyrolysis; samples with high organic matter contents have corresponding high TOC values. This study demonstrates that loss on ignition and thermogravimetry could complement and augment rock evaluation pyrolysis data for petroleum source rock characterization.

## Introduction

**Figure 1:**
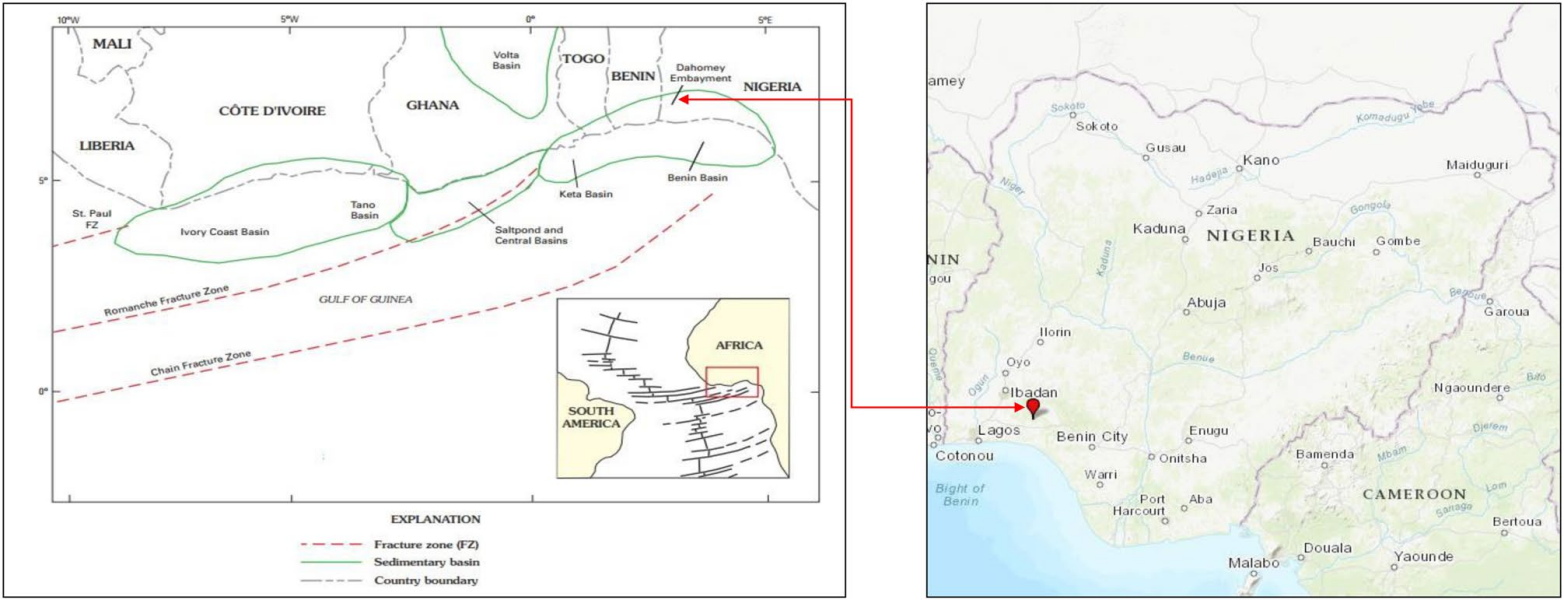
Regional map of a part of West Africa, showing the sedimentary basins and main fracture zones within the Province of the Gulf of Guinea. Mid – Atlantic Ridge and fracture zones are shown in the inset map (Modified from Brownfield and Charpentier^2^). The Red arrows indicate the location of the Eastern Dahomey Basin (Dahomey Embayment) in the regional map and the sample location in southwestern Nigeria.

The Dahomey Basin (Benin Basin), is situated in the province of the Gulf of Guinea (Fig. 1). The region of the Gulf of Guinea comprises wrench-modified^1^ coastal and offshore basins consisting of the Ivory Coast, Tano, Saltpond, Central, Keta, and Benin Basins^2^. These basins evolved in the Mesozoic era by tectonism that was characterized by block and transform faulting during the breakup of African and South American paleocontinents.

The Dahomey Basin extends from southeastern Ghana through Togo and the Republic of Benin to southwestern Nigeria. The western limit of the basin is bounded by the Ghana Ridge, an offset extension of the Romanche Fracture Zone. It is separated from the Niger Delta Basin in the east by a subsurface basement high referred to as the Okitipupa Ridge^3,4^.

The basin hosts a large wedge of Cretaceous to Recent sediments of up to 3,000 m^5–7^, which thicken toward the offshore. The stratigraphy of the eastern sector of the basin (Eastern Dahomey Basin) in southwestern Nigeria, consists of the Cretaceous sediments of the Abeokuta Group (Ise, Afowo, and Araroromi Formations) and the Tertiary sediments of Ewekoro, Akinbo, Oshosun, Ilaro, and Benin Formations. The Afowo Formation of the Abeokuta Group, comprising of sandstones, shales, claystones, and siltstones, has been of great interest owing to reported occurrences of oil sands^5,8–11^.

**Figure 2:**
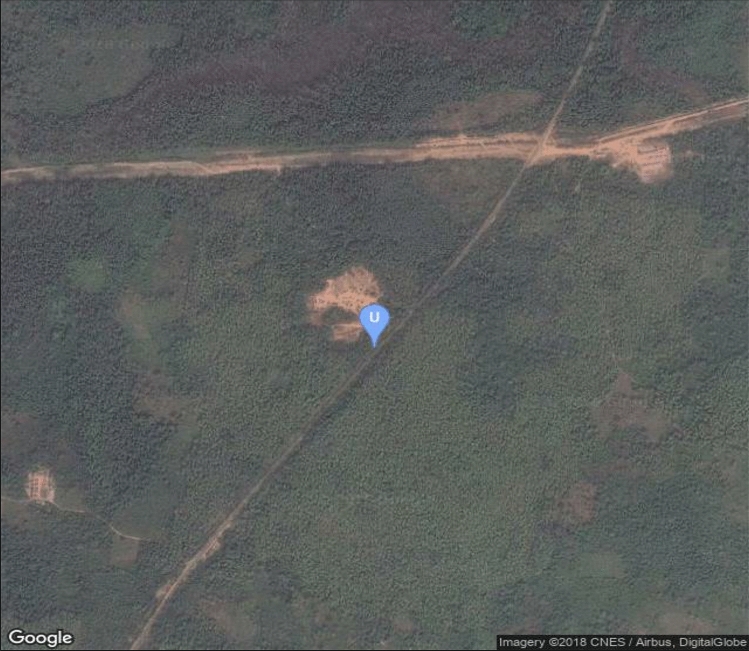
Google map showing the location of the area of study. The outcrop is located within a forestry reserve (J4) in Ogun State, Southwestern Nigeria.

**Figure 3:**
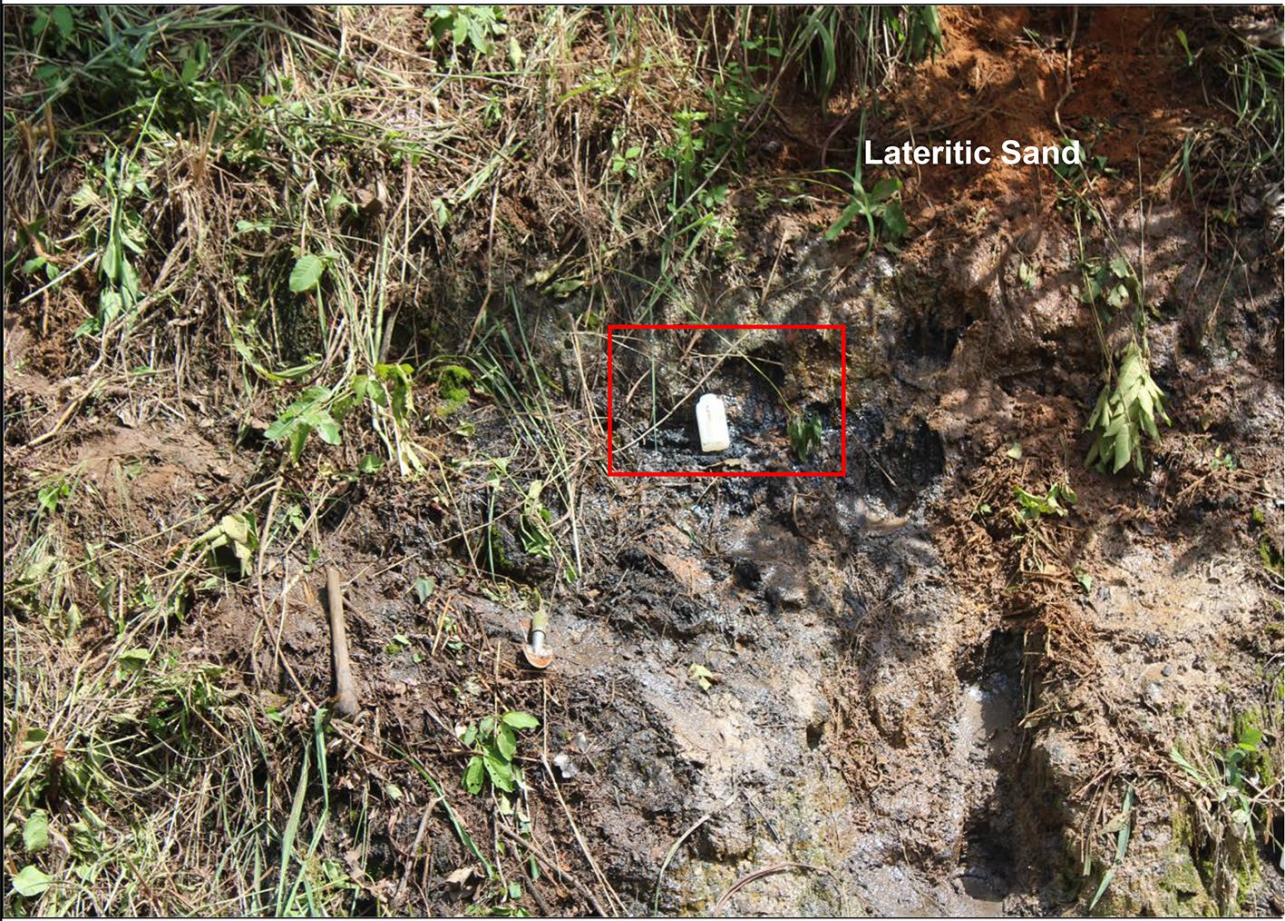
Field photograph showing a section of the outcrop. The outcrop comprises of lateritic sand, oil sand, and claystone. The plastic container displayed in this picture showed a part of the oil sand. The oil sand is underlain by claystone. The lateritic sand at this outcrop is assigned to the Araromi Formation. In contrast, the oilsand and claystone are assigned to the Afowo Formation.

The present study is focused on an outcrop (Figs. 2 and 3) of Afowo Formation located at the latitude 06° 40ʹ 755" N and longitude 04° 18ʹ 613" E at an elevation of 21 m above mean sea level. The lithology at this outcrop consists of oil sand, clay/sand intercalations, and claystone; the oil sand is underlain by claystone. The overarching aim of this study is to evaluate the hydrocarbon generating potential of the claystone using a multi-parameter approach and determine if it is the source rock of the hydrocarbon in the overlying sand.

## Geological setting

The Dahomey (Fig. 1) is a blend of inland/coastal/offshore basin in the Gulf of Guinea that extends from southeastern Ghana through Togo and the Republic of Benin to southwestern Nigeria^3,4^. It is a marginal pull—apart^12^ or marginal sag basin^13^, which evolved during the period of rifting in the Late Jurassic to Early Cretaceous times^7,8,14^. The tectonism was attended by an extended period of thermally induced basin subsidence from the Middle-Upper Cretaceous to Tertiary times as the South American and African tectonic plates entered a drift phase to accommodate the emerging Atlantic Ocean^15,16^. The western limit of the basin is bounded by the Ghana Ridge, an offset extension of the Romanche Fracture Zone^17^, while its eastern boundary is confined by the Benin hinge line, which separates the Okitipupa Structure from the Niger Delta Basin^18^.

The basin hosts a large wedge of Cretaceous to Recent sediments of up to 3,000 m^5,6^, which thicken toward the offshore. The interplay of sedimentation, eustatic sea-level fluctuation of normal faulted graben and horst structure of the basin floor have produced thick accumulations of sediments within the grabens. These accumulations were accompanied by sediment bye-pass and extensive cut and fill structures at or near the shelf margins^19^. The developments resulted in the creation of the essential elements (source rock, reservoir rock, traps, and seals) of a petroleum system in the basin.

The eastern segment of the basin, also referred to as the Eastern Dahomey Basin or Dahomey Embayment, is found in southwestern Nigeria. The stratigraphy of the Eastern Dahomey Basin, summarised in Table 1, has been discussed by various workers, and several classification schemes were proposed^8,20–24^. Despite the classification schemes, age assignments, and terminologies of the different lithological units^5^, within the basin remain mostly controversial. Different stratigraphic names have been proposed for the same formation in different localities and has led to some confusion^20,25^.

**Table 1:** Stratigraphy of the Eastern Dahomey Basin.

Era	Period	Epoch	Stratigraphy of the Eastern Dahomey Basin		
Cenozoic	Quaternary	Holocene	Jones and Hockey^28^	Omatsola and Adegoke^8^	
		Pleistocene			
	Tertiary	Oligocene	Coastal plain sands	Coastal plain sands	
		Eocene	Ilaro formation	Ilaro formation	
				Ososhun formation	
		Paleocene	Ewekoro formation	Akinbo formation	
				Ewekoro formation	
Mesozoic	Cretaceous	Late to early	Abeokuta formation	Abeokuta Group	Araromi formation
					Afowo formation
					Ise formation

The stratigraphic units in the Eastern Dahomey Basin, as defined by Omatsola and Adegoke^8^, consists of the Cretaceous Abeokuta Group and the Tertiary sediments of Ewekoro, Akinbo, Oshosun, Ilaro Formations and the Coastal Plain Sands (Benin Formation).

The Abeokuta Group is further subdivided into Ise, Afowo, and Araromi Formations. Ise Formation is the oldest formation within the Abeokuta Group and rests unconformably on the Crystalline Basement Complex. It comprises of basal conglomerates, medium to coarse-grained sandstones with admixtures of kaolinitic clays. The age is probably Valanginian-Barremian^26^.

The Ise Formation is succeeded by the Afowo Formation. The Afowo Formation is petroliferous, and it is composed of fine to medium and coarse-grained sandstones interbedded with clays, shales, claystones, and siltstones. The sandy facies are hydrocarbon-bearing, while the shales are organic-rich^9^. The lower part of the formation is transitional, with mixed brackish to marginal marine facies that alternate with well-sorted, sub-rounded sand, indicating a littoral or estuarine nearshore depositional environment^22,27^. Based on palynological assemblage, the lower part of the formation was assigned a Turonian age^20^, while the upper part ranges into the Maastrichtian. The formation has also been given a Cenomanian-Coniacian age based on the occurrence of planktonic foraminifera Rotalipora Greenhornensis^26^.

Overlying the Afowo Formation is the Araromi Formation, the youngest Cretaceous sediment in the Eastern Dahomey Basin. The formation is composed of fine to medium-grained sandstone at the base, overlain by shales, siltstones with interbedded limestone, marl, and lignite^5^. The Araromi Formation is fossiliferous and contains abundant planktonic foraminifera, ostracods, pollens, and spores. Based on its fossil contents, Omatsola and Adegoke^8^ assigned it a Maastrichtian age, and it extends into the Paleocene.

The Tertiary sediments of the Eastern Dahomey Basin consists of Ewekoro, Akinbo, Oshosun, Ilaro, and Benin (Coastal Plain Sands) Formations. The Ewekoro Formation overlies the Araromi Formation. It is an extensive limestone body that extends over about 320 km from Ghana in the west to the eastern margin of the basin in southwestern Nigeria^28^. The limestone body is suggested to be of shallow marine origin due to an abundance of coralline algae, gastropods, pelecypods, fragments of echinoid, and other skeletal debris^22^. The formation is assigned Palaeocene.

The Ewekoro Formation is succeeded by Akinbo Formation, which ranges from Paleocene to Eocene. It comprises of shale and clayey sequence^24^ with kaolinite as the dominant clay mineral^29^. The basal part of the formation is defined by bands of glauconite with limestone lenses.

Overlying the Akinbo Formation is the Oshosun Formation of Eocene – Oligocene. The Oshosun Formation consists of greenish-grey or beige clay and shale with interbeds of sandstones; the shale is thickly laminated and glauconitic^5^. The basal beds of the formation ranges in facies from sandstones to mudstones, claystones, clay-shale, or shale^30^. The formation is rich in phosphate^23,31^. The occurrences of fishes and sea snakes in the shale appears to indicate that the formation is of marine origin^26^.

The Ilaro Formation overlies the Oshosun Formation. It comprises of massive, yellowish, poorly consolidated, cross-bedded sandstones^28^ and ranges from Eocene to Oligocene.

The Ilaro Formation is overlain by the Coastal Plain Sands^23^. The Coastal Plain Sands, also referred to as the Benin Formation, is the youngest stratigraphic sequence in the Eastern Dahomey Basin. It consists of poorly sorted sands with clay lenses; the sands are in parts cross-bedded and show transitional to continental characteristics^5^. The formation span Oligocene to Recent.

## Materials and methods

The five claystones that were evaluated for this study were acquired from the outcrop (Fig. 3) of Afowo formation. The outcrop has an overall thickness of 11.65 m, and the lithology is generally composed of 8.42 m of lateritic sand, underlain by 0.79 m of oil sand, and 2.44 m of claystone. The oil sand is saturated with bitumen, and it comprises of sandstone, claystone, and clay/sand intercalations. The claystone samples were acquired at different intervals from 8.7 to 9.9 m with the aid of hand trowel, at an average depth of penetration of 15 cm, inside the outcrop. The samples were then placed in airtight sample bags and carefully labeled. The hand trowel was washed, dried, and wiped with a paper towel before and after acquiring each of the samples to avoid sample contamination.

The evaluation of source rock within any area of study involves the identification and screening analyses of the source rock to assess the quantity and quality of the constituent organic matter, its thermal maturity, and hydrocarbon types that the organic matter type can generate. Rock-Evaluation (Rock–Eval) has been a petroleum industry-standard analysis for source rocks. Still, a multi-parameter approach is recommended since single data types may be in error or affected by contamination^32^. The multi-parameter method requires that more than one analytical method be used to appraise source rocks to establish a high degree of confidence in the results. Thermogravimetric analysis (TGA) and loss on ignition (LOI) could provide valuable information to characterize clay minerals^33^ and to determine total organic carbon^34^. The additional data from loss on ignition and thermogravimetric analysis complement other measurements for robust source rock evaluation.

The source rock samples (claystones) used for this study were assessed with Wildcat Technologies’ HAWK (Hydrocarbon Analyzer with Kinetics) Pyrolysis instrument, Loss on Ignition (utilizing a furnace) and Thermogravimetric Analysis (TGA) using Perkin Elmer STA 6,000. The evaluation was first carried out with Rock–Eval, and the results are incorporated in the present study to assess complementariness and validate findings.

The samples for Rock–Eval, were air-dried to remove any atmospheric moisture content and were then individually ground to a powder with an Agate mortar and pestle. The mortar and pestle were washed, dried, and cleaned with paper towels dosed in methanol before and after each of the samples to avoid sample contamination. A portion of each of the powdered samples was tested for the presence of carbonates, and no acid digestion occurred, indicating the absence of mineral carbon in the source rock.

Sample preparation for loss on ignition and TGA consisted of loading the samples into separate aluminum containers and then drying them in an oven at a temperature of 105 °C for twenty-four hours; they were labeled to aid sample identification. The dried sample was crushed to a fine powder with an Agate mortar and pestle. The mortar and pestle were washed, dried, and wiped with paper towel dosed in methanol to avoid sample contamination.

### Rock–eval pyrolysis

Pyrolysis measurements were carried out using the HAWK method^35^. The pulverized samples were weighed in HAWK crucible individually on a four decimal place balance, and placed in the autosampler tray of the instrument. The crucibles were transferred by the autosampler to the raised HAWK pedestal for placement into the oven. Sample weights of between 10 and 20 mg were used, and two cycles of analyses were performed. The first cycle is pyrolysis, and through programmed temperature heating of up to 650 °C, organic compounds in the analyzed sample were carried by helium from the oven to the flame ionization detector (FID). The FID flame was lit by a mixture of hydrogen and air, resulting in the ionization of the organic compounds; the FID then detected the hydrocarbon constituents. The HAWK oven was cooled down to 300 °C at the end of the first cycle. The second cycle of analysis involved the oxidation of the remaining sample that had already been pyrolyzed. The samples were subjected to programmed heating to a maximum of 750 °C in the presence of dry air, which was used to transport the oxidation products to two infra-red (IR) detectors, one of which detects the CO bonds' bandwidth.

In contrast, the second infrared detector detects the CO2 bonds' bandwidth. The HAWK machine allows for pyrolysis programming to start at temperatures as low as 50 °C or a standby temperature of 100 °C; it ends at a selected maximum that is usually 650 °C. A maximum temperature of 750 °C is adequate when one is only interested in measuring generated hydrocarbons together with total organic carbon (TOC). For carbonate carbon measurements, the oxidation cycle is set to run up to a maximum temperature of 850 °C; inorganic carbonate carbon measurements were not needed for these samples, and the oxidation cycle ended at a temperature of 750 °C.

### Loss on ignition (LOI)

The powdered samples were weighed individually in a crucible on a four decimal place balance and placed in an oven at a temperature of 550 °C for twenty-four hours; the crucibles were labeled to correspond to the individual sample. The samples were then removed from the oven after twenty-four hours, allowed to cool, and then reweighed. The difference in weights provided an estimate of the organic matter content of the samples.

### Thermogravimetric analysis

The pulverized sample was loaded individually with a spatula into the instrument’s sample pan that resides in the furnace; the sample pan is supported by a precision balance that measures the weight of the sample. The spatula was washed, dried, and wiped with a paper towel dosed in methanol before and after loading the sample. The heating of the sample was carried out within a temperature range of 40–700 °C at a rate of 10 °C/min in a nitrogen atmosphere with a purge rate of 20 ml/min.

## Results and discussion

Petroleum originates from the source rock. The viability of a petroleum system depends on its source rock. Without the source rock, all other components (reservoir rock, trap, and seal) and processes needed to exploit hydrocarbon reserves become irrelevant^36^. The present-day geography of petroleum reserves and provinces are controlled mainly by the source rocks^37^. Hydrocarbon generation from the thermal break down of organic matter in the source rock during its burial history is a part of the overall process of thermal metamorphism of organic matter^38^. The hydrocarbon types that are produced by petroleum source rocks throughout its evolutionary pathway are influenced by the quantity/quality of organic matter and organic maturation temperature.

The Afowo clay type was assessed with the loss on ignition and thermogravimetry to determine its organic richness and thermal maturity. The results from loss on ignition and thermogravimetry analysis were validated with Rock–eval data carried out on the same samples in an earlier study by^11^.

**Figure 4:**
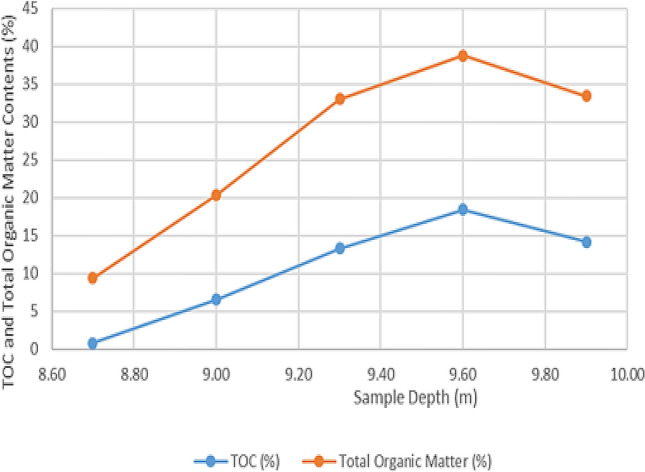
Comparison of Total Organic Carbon (TOC, wt%) and Total Organic Matter Content (TOM, wt%) for five samples of the Afowo clay type evaluated in this study. The sample’s depth positions are shown on the horizontal axis. The changes in TOC and TOM are in sympathy with one another; samples with high TOM contents displayed have high TOC values.

**Figure 5:**
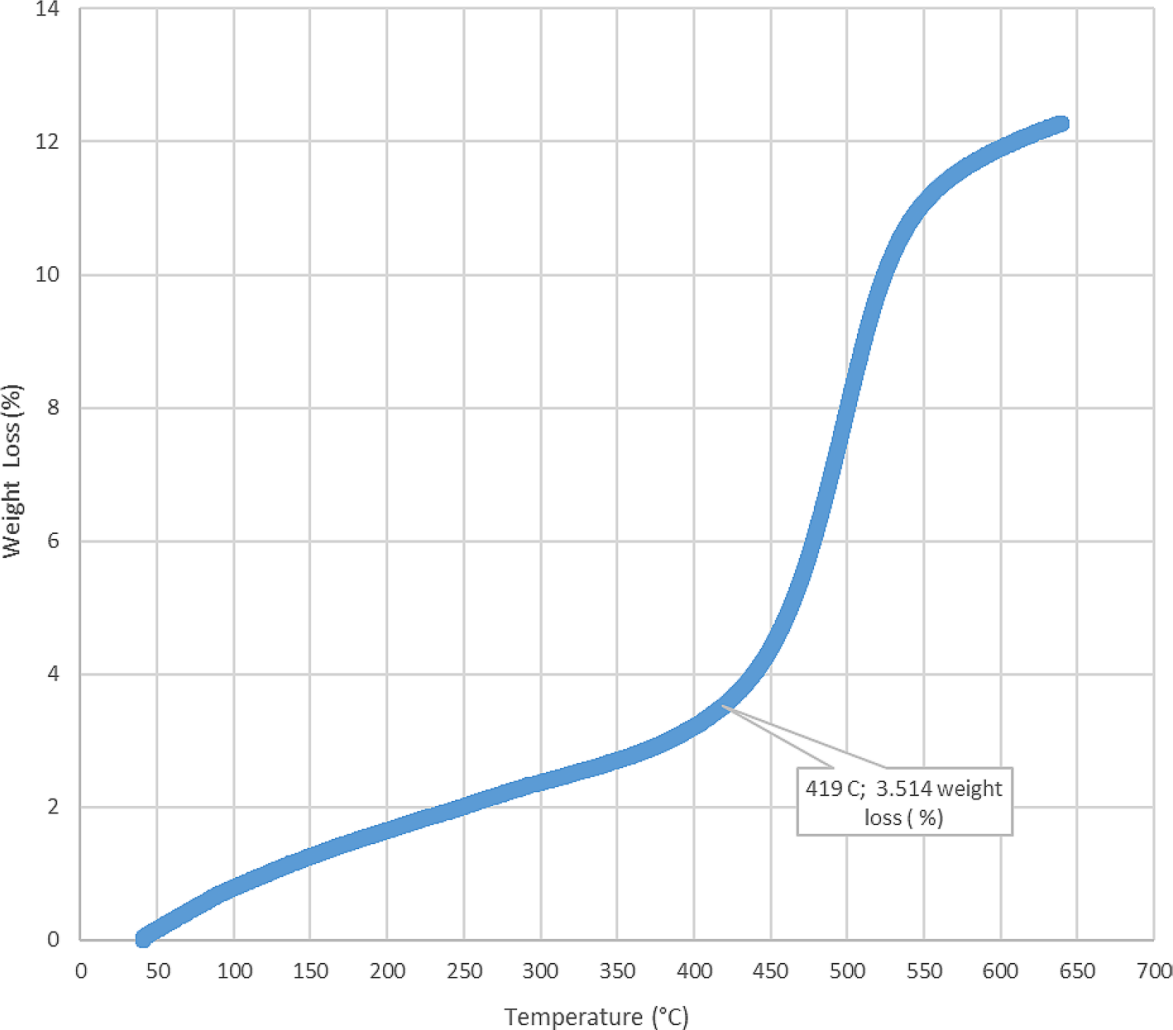
Thermogravimetric analysis (TGA) for Afowo clay sample acquired at a depth of 8.7 m. The inflection point corresponding to the temperature of 419 °C and weight loss of 3.514% is estimated to be the onset temperature at which the maximum generation of hydrocarbon occurred from the pyrolytic decomposition of the organic matter.

**Figure 6:**
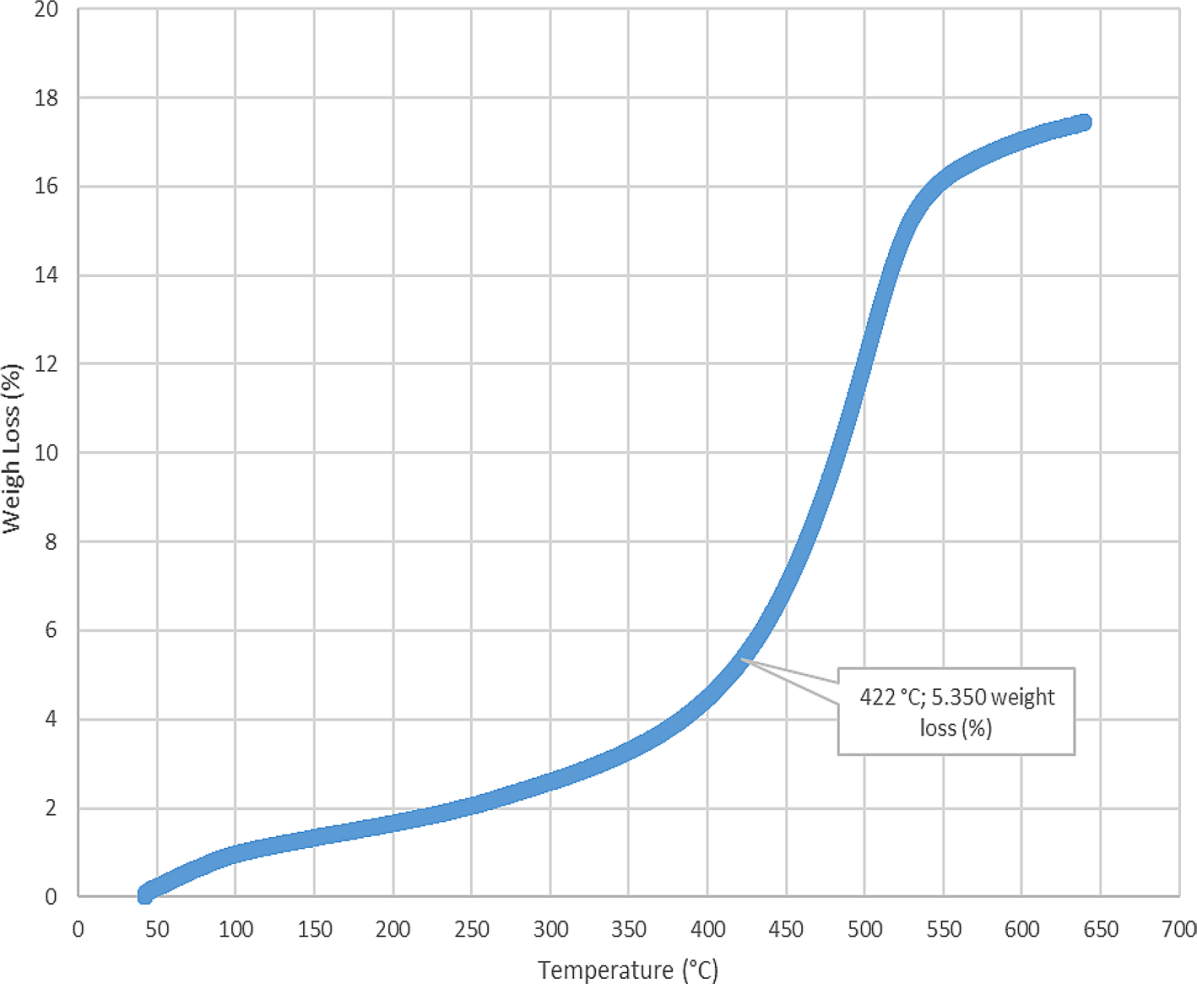
Thermogravimetric analysis (TGA) for Afowo clay sample acquired at a depth of 9.0 m. The inflection point corresponding to the temperature of 422 °C and weight loss of 5.350% is estimated to be the onset temperature at which the maximum generation of hydrocarbons occurred from the pyrolytic decomposition of the organic matter.

**Figure 7:**
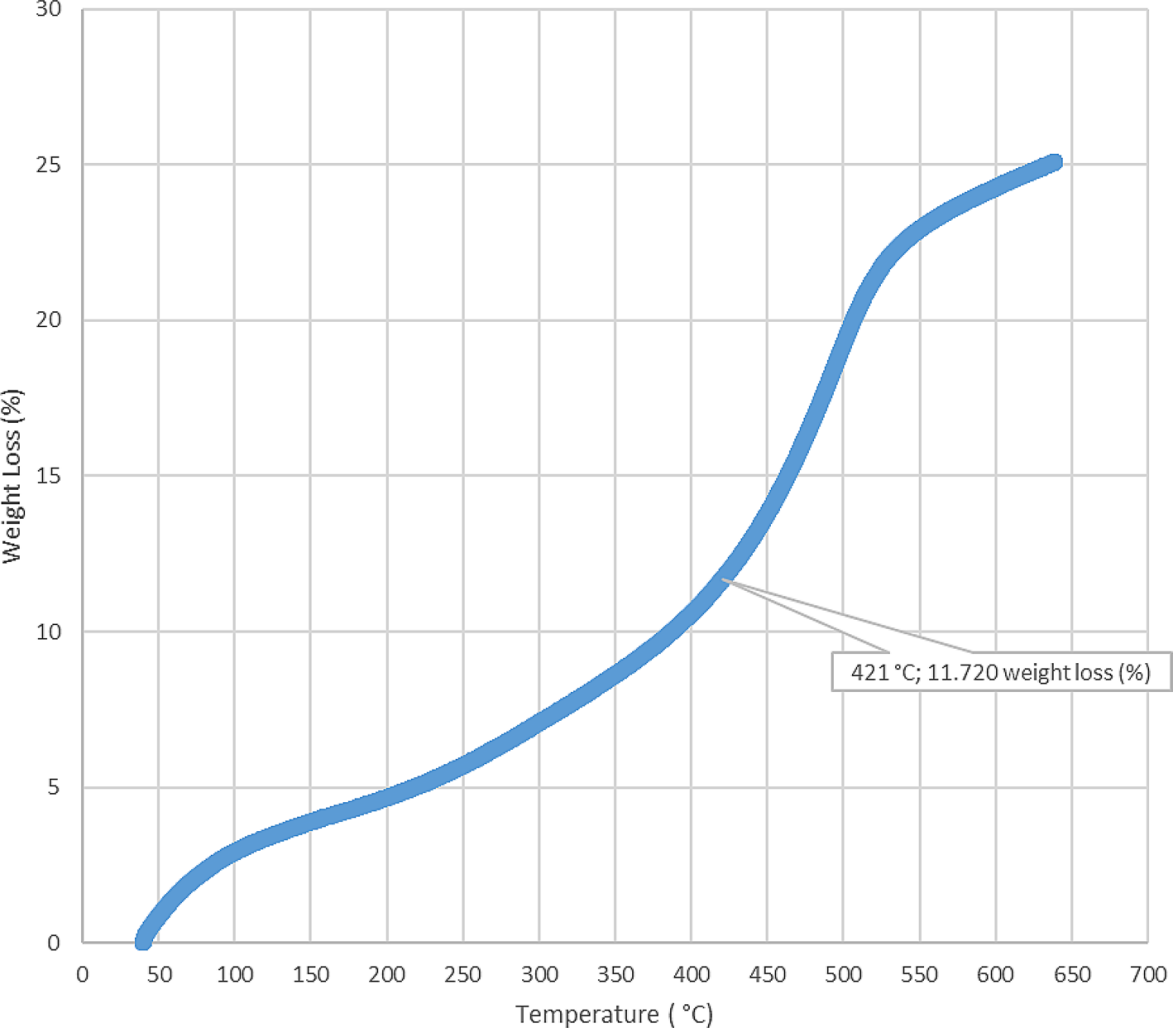
Thermogravimetric analysis (TGA) for Afowo clay sample acquired at a depth of 9.3 m. The inflection point corresponding to a temperature of 421 °C and weight loss of 11.720 (%) is estimated to be the onset temperature at which the maximum generation of hydrocarbon occurred from the pyrolytic decomposition of the organic matter.

**Figure 8:**
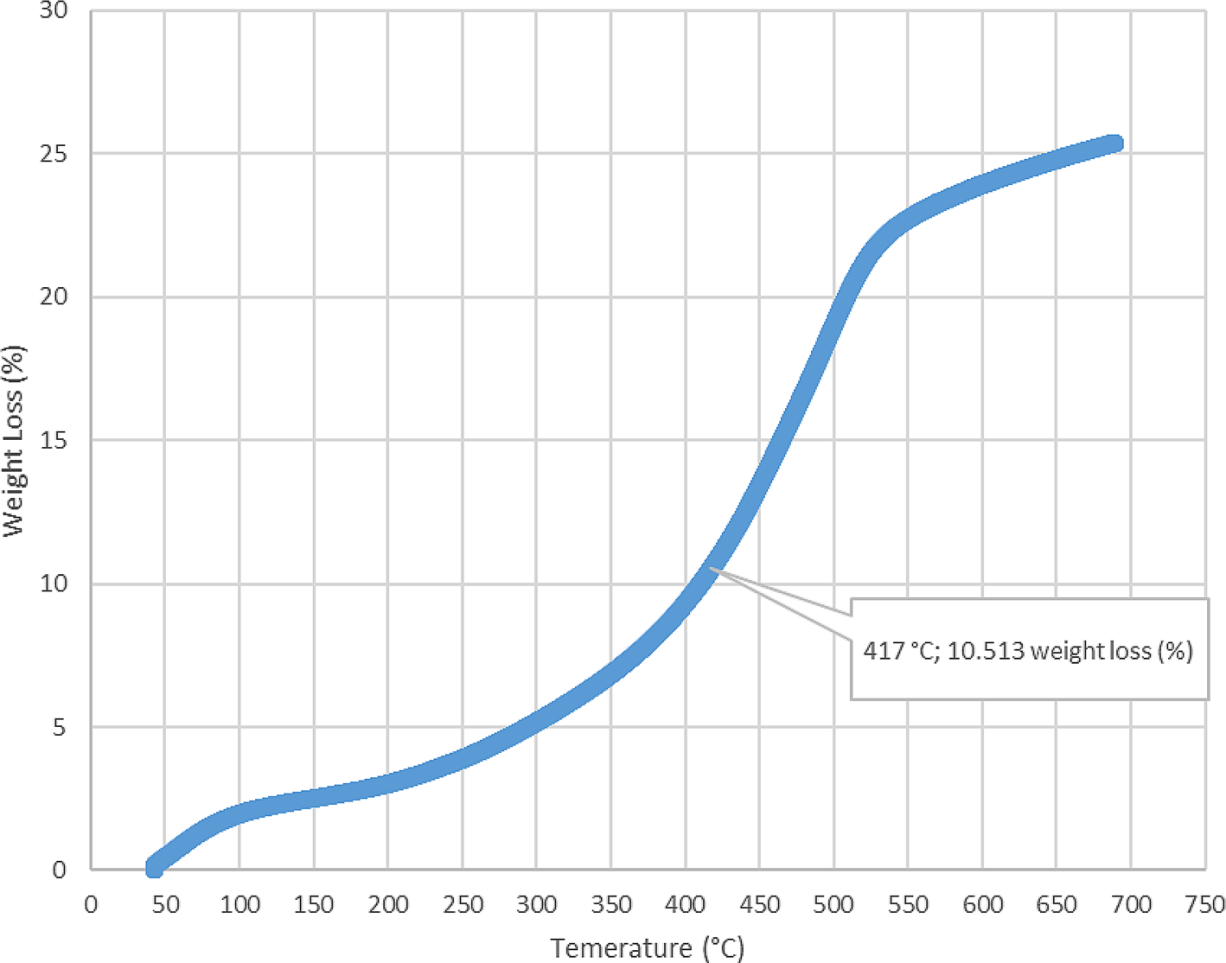
Thermogravimetric analysis (TGA) for Afowo clay sample acquired at a depth of 9.6 m. The inflection point corresponding to a temperature of 417 °C and weight loss of 10.513% is estimated to be the onset temperature at which the maximum generation of hydrocarbons occurred from the pyrolytic decomposition of the organic matter.

**Figure 9:**
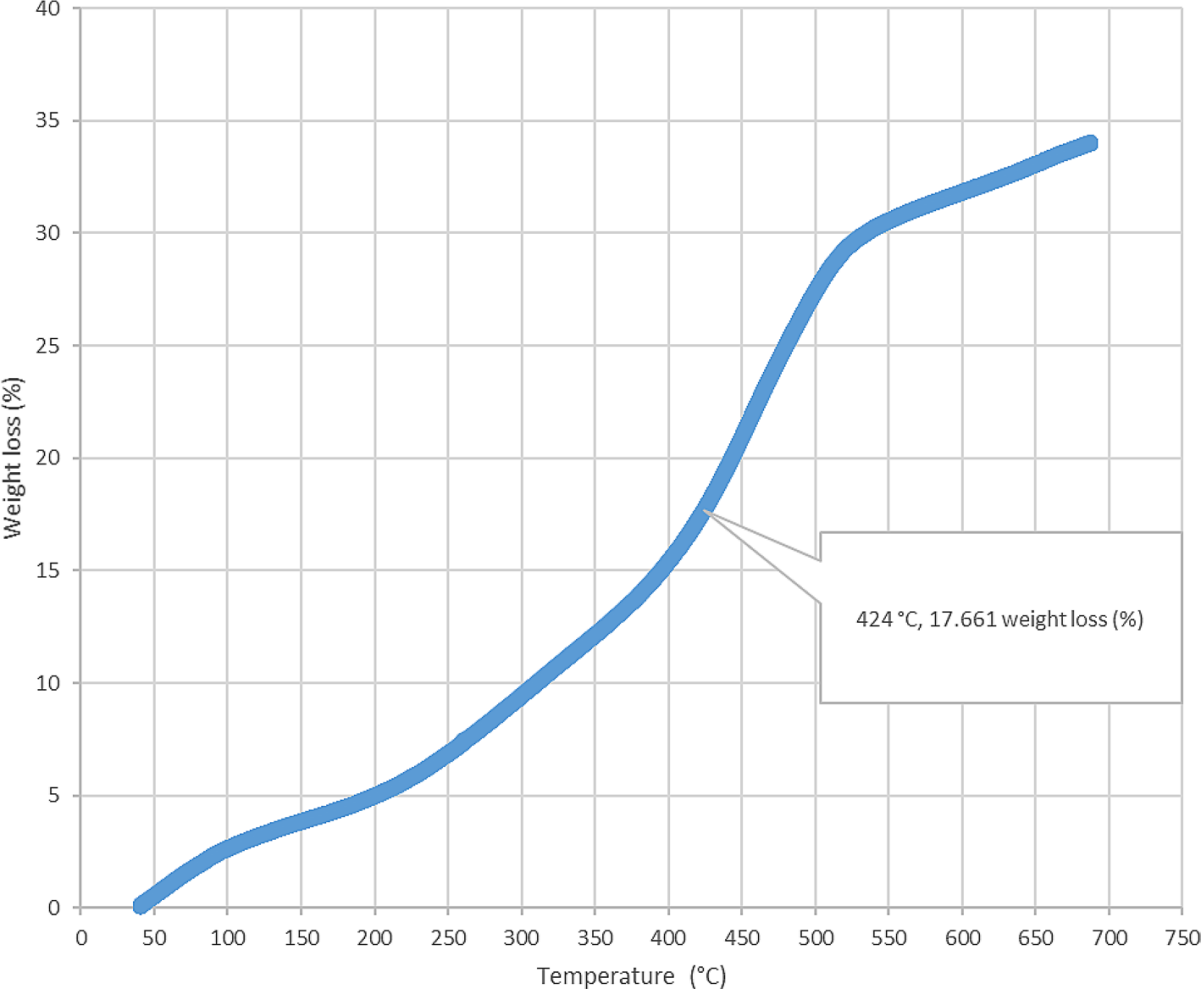
Thermogravimetric analysis (TGA) for Afowo clay sample acquired at a depth of 9.9 m. The inflection point corresponding to the temperature of 424 °C and weight of loss of 17.661 (%) is estimated to be the onset temperature at which the maximum generation of hydrocarbon occurred from the pyrolytic decomposition of the organic matter.

The loss on ignition and thermogravimetric analysis results for the five-sample analyzed in this study are presented in Table 2 and Figs. 4, 5, 6, 7, 8, and 9. The loss on ignition results shown in Table 2 and Fig. 4 indicated an abundance of organic matter. The total organic matter (TOM) content ranged from 9.410 to 38.750 wt%, and it showed a degree of variability with depth. The TOM comprises all the elements that are components of organic matter, such as carbon, hydrogen, nitrogen, and oxygen. It is used as a proxy for organic carbon. A general increase in TOM (Table 2, Fig. 4) from 9.410 to 38.750 wt% was recorded throughout interval 8.7–9.6 m. The TOM content for the sample at a depth of 9.9 m showed a lower value (33.400 wt%) when compared to the preceding TOM (38.750 wt%) at 9.3 m, indicating variation in organic matter richness.

**Table 2:** Loss on ignition (LOI) summary data showing organic matter contents for the Afowo clay type.

Sample ID	Sample depth (m)		Crucible weight (g)	Pre LOI crucible + sample weight (g)	Pre LOI sample weight (g)	Post LOI crucible + sample Weight (g)	Post LOI sample weight (g)	Total organic matter content (TOM, %)
AF-1		8.7	26.963	37.133	10.170	36.176	9.213	9.410
AF-2		9.0	26.387	37.662	11.275	35.373	8.986	20.300
AF-3		9.3	27.790	37.788	9.998	34.490	6.700	32.990
AF-4		9.6	28.112	38.920	10.808	34.732	6.620	38.750
AF-5		9.9	24.760	36.032	11.272	32.267	7.507	33.400

$$Total \,Organic \,Matter=\frac{Pre{\text{-}}ignition \,sample \,weight{\text{-}}Post{\text{-}}ignition \,sample \,weight }{Pre{\text{-}}ignition \,sample \,weight} \times 100.$$

The results obtained from Thermogravimetric Analyses (TGA) are displayed in Figs. 5, 6, 7, 8, and 9. The results indicate organic maturation temperature (Tmax) within the range of 417–424 °C, suggesting that the source rock is thermally immature. The weight loss/gain upon heating the samples in TGA appears to reflect its composition; it ranged from 3.514% to 17.661% at the temperatures that were interpreted as Tmax. The TGA curves (Figs. 5, 6, 7, 8, and 9) showed different curvatures reflecting mineralogy, composition, and decomposition.

**Figure 10:**
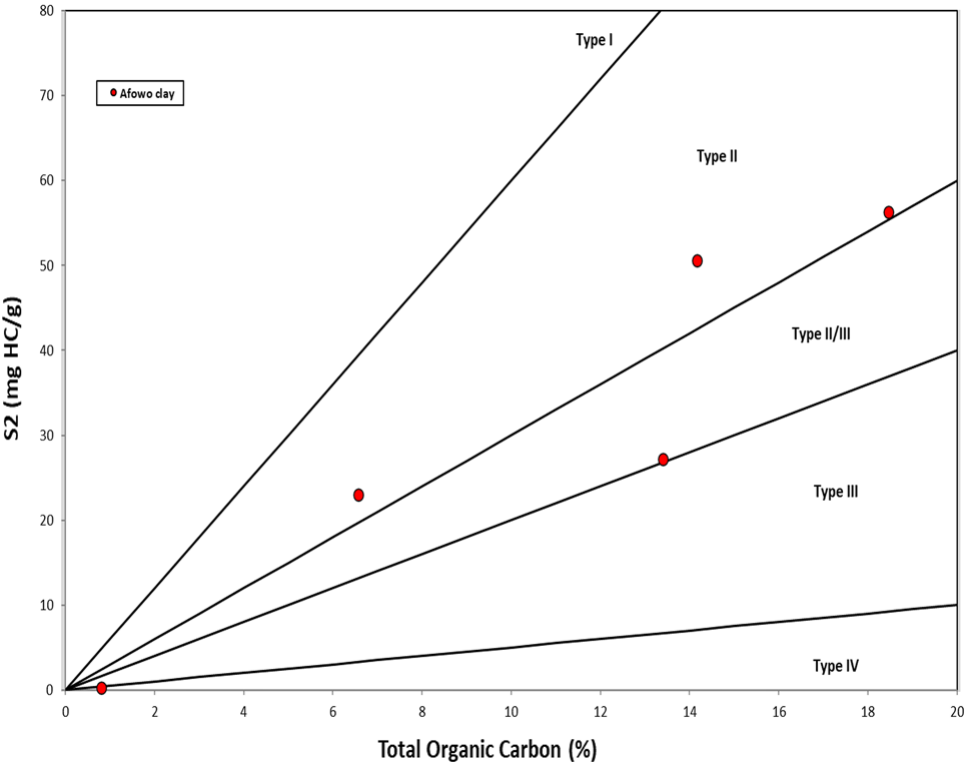
Cross plot of S2 against TOC showing kerogen type. The outlier with almost nil S2 and TOC represented the claystone at a depth of 8.7 m (Adapted from Mohammed et al.^11^). The organic matter types indicated sedimentation in both marine and terrestrial environments.

**Figure 11:**
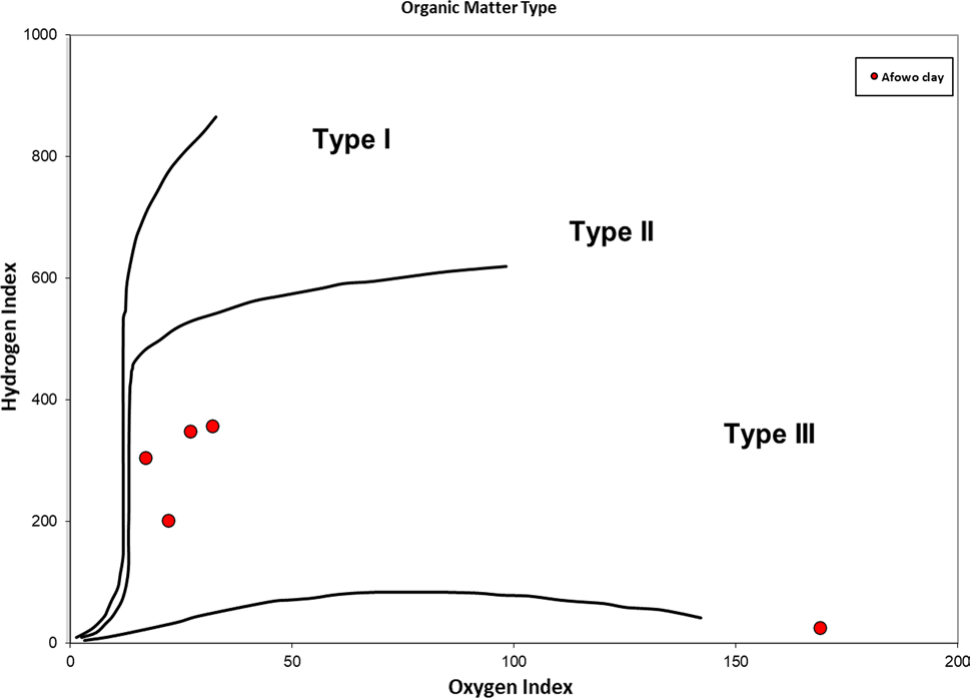
Cross plot of Hydrogen Index versus Oxygen Index. The outlier with very low HI and high OI index represented the claystone at a depth of 8.7 m (Adapted from Mohammed et al.^11^).

**Figure 12:**
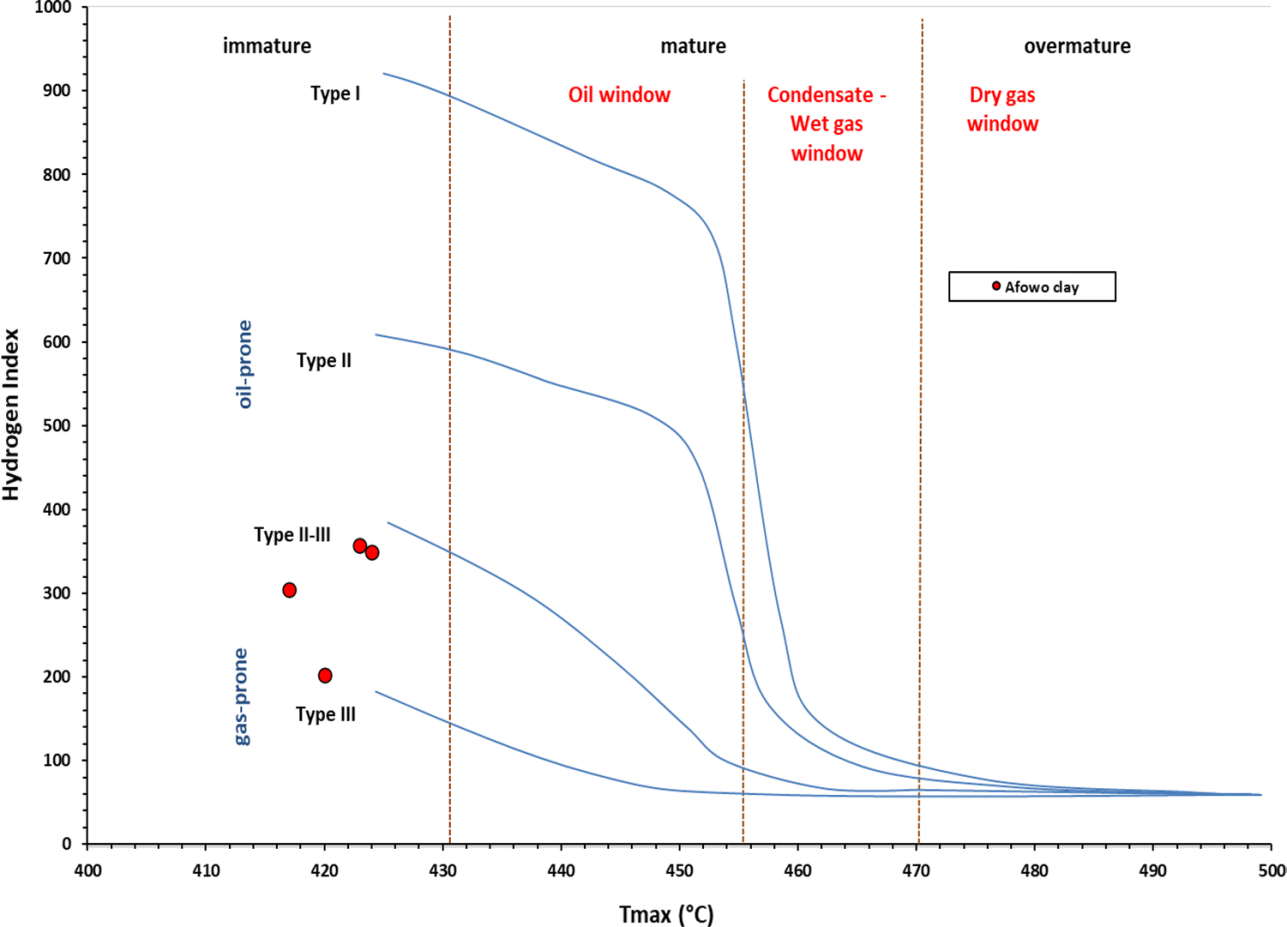
Cross plot of HI against Tmaxshowing kerogen type and organic maturity. It was not possible to estimate Tmaxfor the claystone at a depth of 8.7 m (Adapted from Mohammed et al.^11^). The results suggested that the claystonesare thermally immature.

**Figure 13:**
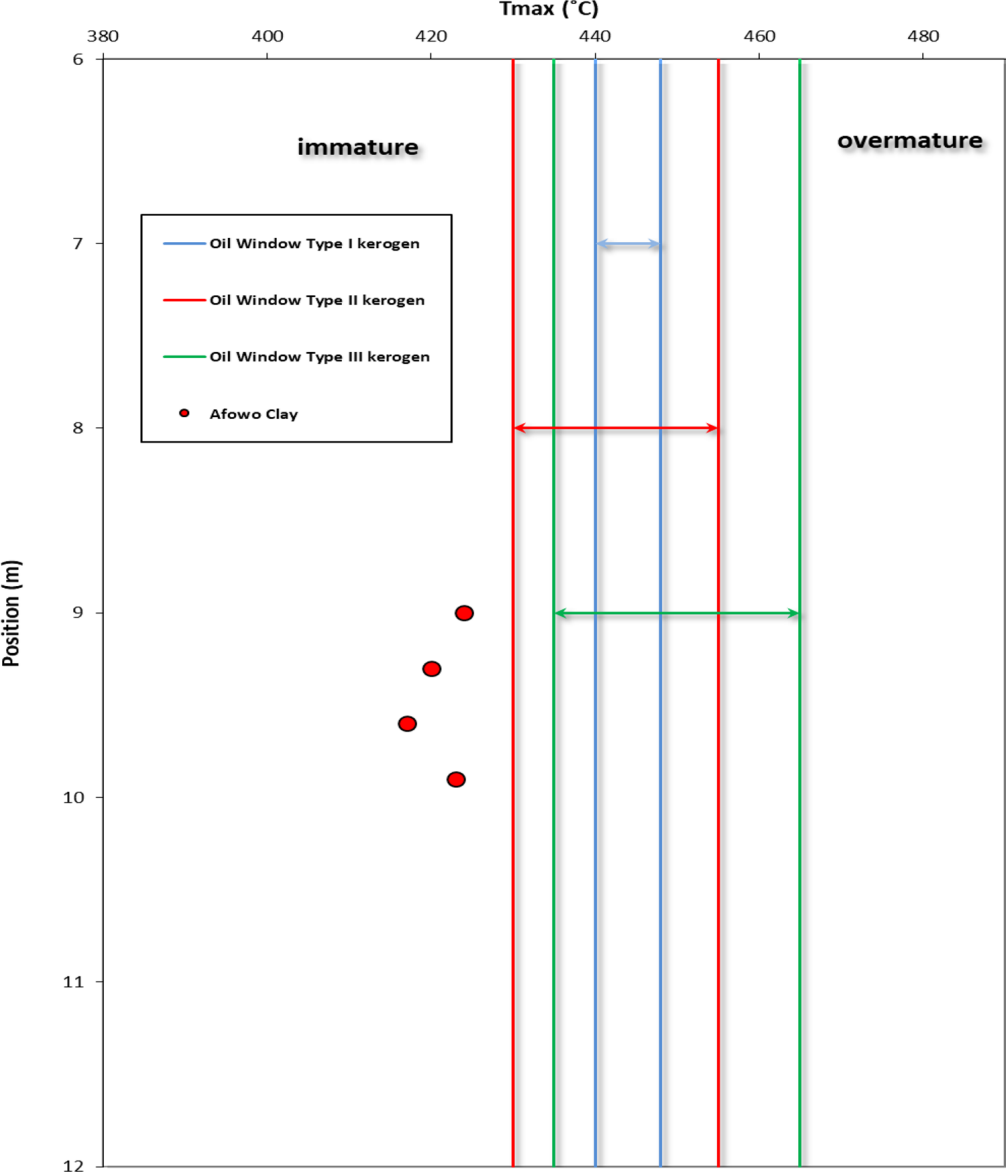
Organic maturation temperature (Tmax) profile for Afowoclay type. The Tmaxfor the samples all showed maturation temperatures below 430 ºC indicating thermal immaturity. It was not possible to obtain Tmaxfor the claystone at a depth of 8.7 m due to lean S2 value (Adapted from Mohammed et al.^11^).

The Rock–Eval results are presented in Table 3 and Figs. 10, 11, 12, and 13. Except for the sample at a depth of 8.7 m, where it was not possible to estimate the Tmax, the pyrolysis results produced critical source rock evaluation parameters. The amount of hydrocarbons generated during the thermal breakdown of kerogen is known as S2, and it indicates the hydrocarbon generating potential remaining in the rock’s kerogen. The S2 for the sample at 8.7 m was lean (0.22 mg HC/g rock). When the S2 peak is little, inaccurate Tmax values are often selected during the Rock–Eval analysis^39^, which complicates the interpretation^40^. The results indicate an abundance of total organic carbon (TOC, wt%) throughout interval 9.0–9.9 m, which ranged from 6.57 to 18.46 wt%. The TOC for the claystone at a depth of 8.7 was lean (0.81 wt%). The S2 ranged from 22.98 mg HC/g rock to 56.33 mg HC/g rock throughout interval 9.0–9.9 m. The variations in TOC and S2 over the depth intervals reflected relative changes in the abundance of organic matter and hydrocarbon generating potential remaining in the kerogen. Both TOC and S2 were used to evaluate the source richness and hydrocarbon generating potential. Except for the sample at a depth of 8.7 m with low TOC and S2 values, the overall results indicate excellent source richness and first-rate hydrocarbon generating potential. The high TOC and S2 of this source rock appears to suggest a low level of thermal maturity; thermal maturation of organic matter mostly parallels a decrease in TOC and S2 due to thermal cracking of the kerogen to generate petroleum. Thermally free hydrocarbon (S1) ranged from 0.18 mg HC/g rock to 10.02 mg Hc/g rock, and their variations appear to correlate with TOC; samples with high TOC values had corresponding high S1.

**Table 3:** HAWK* summary data showing pyrolysis results for samples of the Afowo clays Adapted from Mohammed et al.^11^

Sample ID	Sample depth (m)	Tmax ^o^C	S1 (mg/g)	S2 (mg/g)	S3 (mg/g)	PI	S2/S3	S1/TOC	TOC %	HI	OI
AF-1	8.7	–	0.18	0.22	1.38	0.45	0.16	0.22	0.81	26	169
AF-2	9.0	424	2.85	22.98	1.79	0.11	12.84	0.43	6.57	349	27
AF-3	9.3	420	2.58	27.15	2.96	0.09	9.17	0.19	13.40	202	22
AF-4	9.6	417	10.02	56.33	3.24	0.15	17.39	0.54	18.46	305	17
AF-5	9.9	423	8.21	50.64	4.56	0.14	11.11	0.58	14.17	357	32

It was not possible to estimate Tmax for the clay sample at a depth of 8.7 m owing to its lean hydrocarbon generating potential.

S1 = The amount of free hydrocarbon in the sample before the analysis in mg HC/g rock.

S2 = The amount of hydrocarbon generated during thermal pyrolysis of the sample in mg Hc/g rock.

S3 = The carbon dioxide yield during thermal breakdown of kerogen in mg CO_2_/g rock.

PI = Production Index.

TOC = Total organic carbon in wt.%

HI = Hydrogen index.

OI = Hydrogen Index.

The cross plots of S2 against TOC (Fig. 10), hydrogen index versus oxygen index (Fig. 11), and hydrogen index against Tmax (Fig. 12), provided indications of source quality. The plots suggest Type II, and Type II/III kerogens for source rocks over the interval 9.0–9.9 m. These kerogen types have the potentials to generate oil and gas at maturity. In contrast, the results indicate Type IV kerogen for the source rock at a depth of 8.7 m; Type IV kerogen has nil potential for hydrocarbon generation.

Tmax is the principal Rock–Eval parameter to assess the thermal maturity of kerogen. Figure 13 shows the variation in Tmax with depth. The Tmax values ranged from 417 to 424 °C, indicating thermal immaturity.

**Figure 14:**
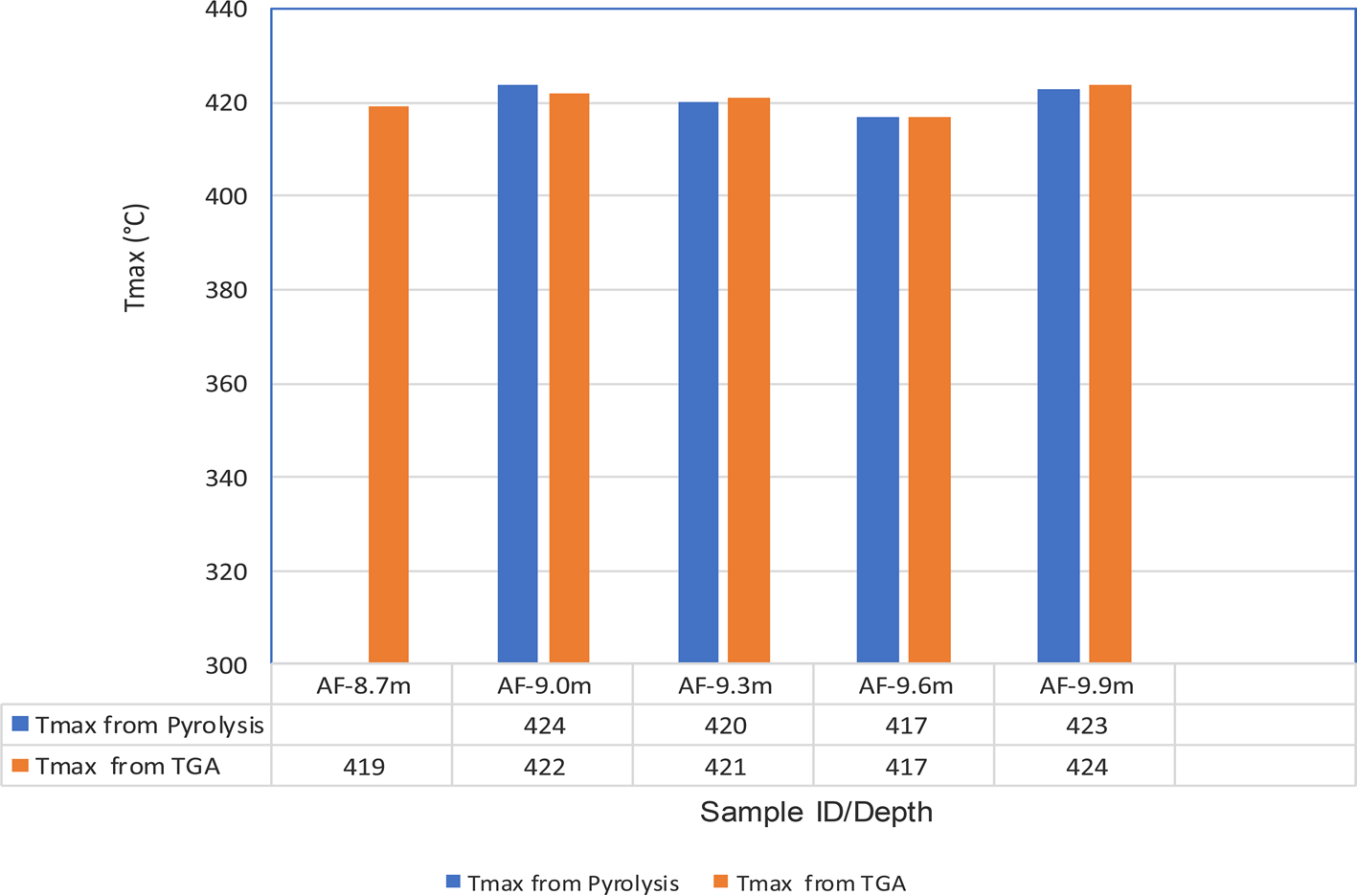
Comparison of organic maturation temperature (Tmax) from rock-evalpyrolysis and thermogravimetricanalysis. The results indicated compatible Tmaxfrom the different measurements. While it was not possible to obtain Tmax from rock-eval pyrolysis for the claystone at a depth of 8.7 m, reliable Tmax was estimated for the same sample with the aid of thermogravimetry.

The analytical results from loss on Ignition (LOI) and thermogravimetry presented in Table 2 and Figs. 4, 5, 6, 7, 8, and 9 supported the geochemical data from rock–eval pyrolysis. The total organic matter (Table 2) is distinct and separate from total organic carbon; it comprises of other elements that are components of organic matter, which includes organic carbon. The TOM results suggest a rich source rock. The trends of total organic matter contents (TOM, wt%) and total organic carbon (TOC, wt%) from loss on ignition and rock–Eval pyrolysis implied positive correlation (Fig. 4). Samples with high TOM contents have corresponding high TOC. The Tmax values from TGA (Figs. 5, 6, 7, 8, and 9) showed a range of 417–424 °C, which is similar to Tmax estimated from rock–eval (Fig. 14); it was possible to assess Tmax for the sample at 8.7 m from thermogravimetric analysis.

## Conclusions

A multi-parameter approach encompassing Rock–Eval, Thermogravimetric Analysis, and Loss on Ignition was used to evaluate the source rock potential of the Afowo clay type. The results showed that the claystones have excellent source richness with excellent potential to generate oil and gas at maturity.

The observations and conclusions from these results are:

1. Thermogravimetric and loss on ignition analyses showed that the Afowo clay type is rich in organic matter contents and are thermally immature.
2. The rock–eval results indicated excellent source richness for the same Afowo clay type samples throughout interval 9.0–9.9 m. The Tmax suggests that the source rock is immature. These results are compatible with the findings from loss on ignition and thermogravimetric analysis.
3. It was possible to determine Tmax by thermogravimetric analysis for the source rock sample at a depth of 8.7 m. The constraint of lean S2 for this sample made it challenging to assess its Tmax using Rock–Eval.
4. The total organic matter contents from loss on ignition (LOI) and Tmax from thermogravimetric analysis (TGA), all supported the geochemical data from rock–eval. Both LOI and TGA can enhance a multi-parameter approach to evaluate and characterize petroleum source rocks.
5. The results from both Rock–Eval and TGA showed that the claystones are thermally immature and could not have been the source of the hydrocarbons in the overlying sand. The hydrocarbons in the overlying sand (oil sand) were possibly generated by different source rocks within the basin, where they attained a greater burial depth and temperature.

## References

[1] Clifford AC (1986). African oil: past, present, and future. AAPG Mem. (Am. Assoc. Pet. Geol.).

[2] Brownfield, M. E. & Charpentier, R. E. Geology and total petroleum systems of the Gulf of Guinea Province of West Africa. *USGS Bull.* 32 (2006).

[3] Obaje NG (2009). Geology and Mineral Resources of Nigeria.

[4] Oli IC, Okeke OC, Abiahu CMG, Anifowose FA, Fagorite VI (2019). A review of the geology and mineral resources of Dahomey Basin, Southwestern Nigeria. Int. J. Environ. Sci. Nat. Resour..

[5] Nton ME, Ikhane PR, Tijani MN (2009). Aspect of rock-eval studies of the Maastrichtian-Eocene sediments from subsurface, in the eastern Dahomey basin southwestern Nigeria. Eur. J. Sci. Res..

[6] Ogala JE (2019). Geochemical and organic petrological study of bituminous sediments from Dahomey Basin, SW Nigeria. Mar. Pet. Geol..

[7] Whiteman AJ (1982). Nigeria: Its Petroleum Geology, Resources and Potential.

[8] Omatsola ME, Adegoke OS (1981). Tectonic evolution and cretaceous stratigraphy of the Dahomey Basin. Niger. J. Min. Geol..

[9] Enu EI, Ako BD, Enu EI (1990). Nature and occurrence of tar sands in Nigeria. Occurrence, Utilization and Economics of Tar Sands.

[10] Ndukwe VA, Ogunyinka BO, Abrakasa S (2015). Some aspect of the petroleum geochemistry of tar sand deposits in western Nigeria. Pyrex J. Geol. Min. Res..

[11] Mohammed S, Opuwari M, Titinchi S, Bata T, Mohammed BA (2019). Evaluation of source rock potential and hydrocarbon composition of oil sand and associated clay deposits from the Eastern Dahomey Basin, Nigeria. J. Afr. Earth Sci..

[12] Klemme HD, Fischer AG (1975). Geothermal gradient, heat flow and hydrocarbon recovery. Petroleum and Global Tectonics.

[13] Kingston DR, Dishroon CP, Williams PA (1983). Global basin classification system. AAPG Bull. (Am. Assoc. Pet. Geol.).

[14] Adekeye OA, Akande SO, Adeoye JA (2019). The assessment of potential source rocks of Maastrichtian Araromi formation in Araromi and Gbekebo wells Dahomey Basin, southwestern Nigeria. Heliyon.

[15] Kaki C, d’Almeida GAF, Yalo N, Amelina S (2013). Géologie et système pétrolier du bassin offshore du Benin (Benin). Oil Gas Sci. Technol..

[16] Storey BC (1995). The role of mantle plumes in continental breakup: Case Histories from Gondwanaland. Nature.

[17] Olabode SO (2015). Subsidence patterns in the Nigerian sector of Benin (Dahomey) Basin: evidence from three offshore wells. Ife J. Sci..

[18] Akinmosin A, Omosanya KO, Olawole AO (2015). Hydrocarbon potential of some Afowo shale deposits in part of South Western Nigeria. Int. J. Afr. Asian Stud..

[19] Ministry of Mines and Steel Development (MMSD). *Tarsands & Bitumen Investment Opportunities in Nigeria*. (2009).

[20] Billman HG (1992). Offshore stratigraphy and paleontology of the dahomey embayment, West African. Niger. Assoc. Pet. Explor. Bull..

[21] Coker SJ, Ejedawe JE, Oshiorienua JA (1983). Hydrocarbon source potentials of Cretaceous rocks of Okitipupa Uplift, Nigeria. Niger. J. Min. Geol..

[22] Elueze AA, Nton ME (2004). Organic geochemical appraisal of limestone and shales in part of eastern Dahomey Basin, southwestern Nigeria. Niger. J. Min. Geol..

[23] Jones HA, Hockey RD (1964). The geology of part of southwestern Nigeria. Geol. Surv. Niger. Bull..

[24] Ogbe, F. G. A. Stratigraphy of strata exposed in the Ewekoro quarry, Western Nigeria. In *African Geology* 305–322 (University Press, Ibadan, 1972).

[25] Coker, S. J. L. Field excursion guide to tar sand outcrops. (2002).

[26] d’Almeida GAF, Kaki C, Adeoye JA (2016). Benin and Western Nigeria Offshore Basins: a stratigraphic nomenclature comparison. Int. J. Geosci..

[27] Nwajide CS (2013). Geology of Nigeria’s Sedimentary Basins.

[28] Jones HA, Hockey RD (1964). The geology of part of Southwestern Nigeria. Bull. Geol. Surv. Nig..

[29] Nton ME, Eze FP, Elueze AA (2005). Aspects of Source rock evaluation and diagenetic history of the Akinbo shale, Eastern Dahomey Basin, Southwestern Nigeria. Niger. Assoc. Pet. Explor..

[30] Okosun EA (1998). Review of the early tertiary stratigraphy of southwestern Nigeria. Niger. J. Min. Geol..

[31] Nton ME (2001). Sedimentological and Geochemical Studies of Rock Units in the Eastern Dahomey Basin, Southwestern Nigeria.

[32] Dembicki HJ (2017). Practical Petroleum Geochemistry for Exploration and Production.

[33] Jozanikohan G, Sahabi F, Norouzi GH, Memarian H (2015). Thermal analysis: a complementary method to study the Shurijeh Clay minerals. IJMGE.

[34] Al-Selwi A, Joshi M (2015). Source rock evaluation using total organic carbon (TOC) and the loss-on-ignition (LOI) techniques. Oil Gas Res..

[35] Maende A (2014). Application Note (181003–2) HAWK Analysis for Drill Cuttings, Cores, Outcrop and Soil Samples, Classical Pyrolysis and HAWK Petroleum Assessment Method^TM^ (HAWK-PAM).

[36] McCarthy K, Rojas K, Niemann M, Palrnowski D, Peters K, Stankiewicz A (2011). Basic petroleum geochemistry for source rock evaluation. Oilf. Rev..

[37] Peters KE, Walters CC, Moldowan JM (2005). The Biomarker Guide Volume 1.

[38] Tissot BP, Welte DH (1984). Petroleum Formation and Occurrence.

[39] Peters KE (1986). Guidelines for evaluating petroleum source rock using programmed pyrolysis. Am. Assoc. Pet. Geol. Bull..

[40] Dembicki H (2009). Three common source rock evaluation errors made by geologists during prospect or play appraisals. Am. Assoc. Pet. Geol. Bull..

